# Microbiome as a biomarker and therapeutic target in pancreatic cancer

**DOI:** 10.1186/s12866-023-03166-4

**Published:** 2024-01-05

**Authors:** Ghazaleh Pourali, Danial Kazemi, Amir Shayan Chadeganipour, Mahshid Arastonejad, Sara Naghizadeh Kashani, Roozbeh Pourali, Mina Maftooh, Hamed Akbarzade, Hamid Fiuji, Seyed Mahdi Hassanian, Majid Ghayour-Mobarhan, Gordon A. Ferns, Majid Khazaei, Amir Avan

**Affiliations:** 1 Metabolic Syndrome Research Center, Mashhad University of Medical Sciences, Mashhad, Iran; 2 Student Research Committee, Isfahan University of Medical Sciences, Hezar Jerib Street, Isfahan, Iran; 3 Department of Human and Molecular Genetics, Virginia Commonwealth University, Richmond, VA USA; 4 Student Research Committee, Faculty of Veterinary Medicine, Ferdowsi University of Mashhad, Mashhad, Iran; 5 Basic Sciences Research Institute, Mashhad University of Medical Sciences, Mashhad, Iran; 6 Brighton & Sussex Medical School, Department of Medical Education, Falmer, Brighton, Sussex, BN1 9PH UK; 7 College of Medicine, University of Warith Al-Anbiyaa, Karbala, Iraq; 8 School of Mechanical, Medical and Process Engineering, Science and Engineering Faculty, Queensland University of Technology, 2 George St, Brisbane City, QLD 4000 Australia; 9 School of Medicine, Isfahan University of Medical Sciences, Hezar Jerib Street, Isfahan, Iran

**Keywords:** Microbiome, Pancreatic cancer, Biomarker, Treatment, Probiotics, Diagnosis, Prognosis

## Abstract

Studying the effects of the microbiome on the development of different types of cancer has recently received increasing research attention. In this context, the microbial content of organs of the gastrointestinal tract has been proposed to play a potential role in the development of pancreatic cancer (PC). Proposed mechanisms for the pathogenesis of PC include persistent inflammation caused by microbiota leading to an impairment of antitumor immune surveillance and altered cellular processes in the tumor microenvironment. The limited available diagnostic markers that can currently be used for screening suggest the importance of microbial composition as a non-invasive biomarker that can be used in clinical settings. Samples including saliva, stool, and blood can be analyzed by 16 s rRNA sequencing to determine the relative abundance of specific bacteria. Studies have shown the potentially beneficial effects of prebiotics, probiotics, antibiotics, fecal microbial transplantation, and bacteriophage therapy in altering microbial diversity, and subsequently improving treatment outcomes. In this review, we summarize the potential impact of the microbiome in the pathogenesis of PC, and the role these microorganisms might play as biomarkers in the diagnosis and determining the prognosis of patients. We also discuss novel treatment methods being used to minimize or prevent the progression of dysbiosis by modulating the microbial composition. Emerging evidence is supportive of applying these findings to improve current therapeutic strategies employed in the treatment of PC.

## Introduction

Pancreatic cancer (PC) is reported to be the third most common cause of cancer-related deaths in both sexes in the USA, with a very low 5-year survival rate of 12%, because most patients are identified in the late stages of disease [1]. It is estimated that there will be 64,050 new cases and 50,550 deaths due to PC in 2023 [1]. The high mortality associated with PC increases the importance of identifying new diagnostic markers and therapies to enable the initiation of early intervention [2]. The most prevalent form of PC is pancreatic ductal adenocarcinoma (PDAC) [3]. Tumor growth is triggered by mutations and subsequent inactivation of tumor suppressor genes that cooperate with *KRAS* oncogene mutations [3]. PDAC is a highly aggressive cancer, and currently, only approximately 15–20% of patients have tumors that are suitable for surgical resection, offering a chance of potential cure. However, surgical intervention is a highly invasive procedure, and even after successful resection, the 5-year survival rate remains limited, reaching only 20% [4].

In addition to genetic alterations, the tumor microenvironment is likely to play a pivotal role in the pathogenesis of PC [5]. Non-neoplastic cells, including endothelial cells, immune cells, and fibroblasts, interact with PDAC cells and may determine tumor growth and the effectiveness of therapy [6]. The microbiota can stimulate persistent inflammation, causing alterations in the antitumor immune system, leading to changes in cellular metabolism in the tumor microenvironment [7, 8]. As a result, the microbiota is able to greatly influence the prognosis of malignancies and affect therapeutic efficacy in some patients [9]. There is still no single screening procedure recommended for PC diagnosis that is applicable to the entire population [10–13]. Based on international guidelines, MRI/MRCP and endoscopic ultrasound plus fasting glucose or HbA1C, monitoring new-onset diabetes, should be performed on patients with an elevated risk of familial pancreatic cancer (FPC). These guidelines aim to identify Stage I pancreatic cancer and high-grade dysplastic precursor lesions, namely pancreatic intraepithelial neoplasms (PanINs) and intraductal papillary mucinous neoplasms (IPMNs) [14]. Currently, screening tests are only suggested for family members of PC patients with a higher chance of being affected by FPC [15]. A window of opportunity exists before the manifestation of clinical symptoms, during which the detection of precursor lesions offers a chance for preventive measures to hinder invasiveness. To enhance opportunities for potential treatment, early detection is essential [16]. Lowering the detection limit can be achieved through the integration of novel diagnostic tests and methods. Given the widespread presence and significance of the GI microbiota, monitoring this aspect within the GI holds promise for enhancing outcomes in future patients.

Several novel biomarkers and techniques have been introduced to diagnose cancers including PC at an earlier stage of the disease, including serum biomarkers, imaging techniques, genetic testing, and identification of high-risk premalignant lesions [17, 18]. The gastrointestinal (GI) tract and pancreas have a continuous ductal structure [19]. The composition of the microbiota in each of these segments of the GI tract has the potential to influence the other and give rise to diseases resulting from abnormally abundant pathogens. 16S rRNA sequencing is the most common approach to identify the diversity and distribution of microbial communities in various reservoirs of the human body [20, 21]. The functional properties of microbiota samples from patients are studied using metagenomic, metaproteomic, and metabolomic methods [22].

Dysbiosis refers to changes in diversity of the microbial population, that is accompanied by alterations in the taxonomic microbial profiles [23, 24]. There is good evidence for the possible role of dysbiosis in the pathogenesis of various GI tract pathologies, including PC [25–28]. Various sequencing techniques have been used to investigate the importance of different microbial species in the formation and progression of GI tract pathologies and neoplasms [29]. Research on the effects of microbial diversity on PC is still in early stages, but emerging evidence provides a link between the microbiome and PC [30, 31]. Various bacterial, fungal, and viral species exist in the GI tract and may be involved in the development of PC. The microbiota partially exerts its effects by modulation of tumor microenvironment, which may also lead to alter treatment efficacy [32–34].

In the present review, we summarize the role that different bacterial, viral, and fungal species may play in the pathogenesis and development of PC. The effects of microbial diversity and dysbiosis on the pathogenesis and effects on therapy and management of the disease are also summarized. Additionally, we present the possible clinical applications of these microbial species as prognostic and diagnostic biomarkers. We also discuss some of the therapeutic methods that may be used to treat PC by influencing GI tract microbial diversity, including bacteriophage therapy, probiotics, antibiotics, and fecal microbial transplantation.

### Metagenomics-based approaches for community characterization

Identification and characterization of biomarkers play a crucial role in improving the early detection, diagnosis, and management of PC [35]. Several techniques and approaches have been employed to identify and validate biomarkers associated with PC.

Studies have utilized techniques such as 16S rRNA gene sequencing and metagenomic shotgun sequencing to characterize the composition and diversity of the gut microbiota in PC patients [36]. 16S rRNA gene sequencing is used to analyze the microbial diversity present in the pancreatic tumor and surrounding tissues [37]. 16S rRNA genes have been found to be highly conserved and are used for taxonomic classification, serving as a basis for accurate characterization techniques such as gene sequencing and amplification [38]. The most recent development in characterizing the gut microbiota is metagenomics which is the study of the genetic material acquired directly from clinical or environmental samples [39]. Metagenomics aid in investigation of the collective genomes of the environment, and crosstalk between microbial components and disease formation to determine causative mechanisms [40]. Metagenomic sequencing involves direct DNA sequencing of the microbial communities present in the pancreatic tumor. It identifies the functional characteristics of the microbiome that are associated with PC [41]. Currently, samples for next-generation sequencing are collected from feces, mucosal biopsy, and intestinal aspiration. However, these methods are not completely accurate reflections of intestinal microbiota composition [42]. Metabolomics is the study of small molecules (metabolites) produced by the microbiome. It can help in identifying specific metabolites as biomarkers for PC diagnosis and prognosis [43]. These approaches have revealed potential associations between specific bacterial taxa, dysbiosis, and PC development [44]. Furthermore, metagenomic and metatranscriptomic analyses have provided insights into the functional properties of the microbiome and its potential impact on PC pathogenesis [10]. Computational methods are used to analyze the microbiome data obtained from sequencing [45, 46]. These methods include machine learning algorithms, statistical models, and network analysis methods that help in identifying microbial biomarkers for cancer [45]. The identification and characterization of biomarkers are critical for improving the early detection, diagnosis, and management of PC. Employing techniques such as 16S rRNA gene sequencing, metagenomics, and metabolomics has provided valuable insights into the composition, diversity, and functional characteristics of the gut microbiota in PC patients. Metagenomics, particularly, has facilitated the investigation of the collective genomes of the environment and the interplay between microbial components and disease formation.

### The role of the microbiome in the development of pancreatic cancer

**Fig. 1:**
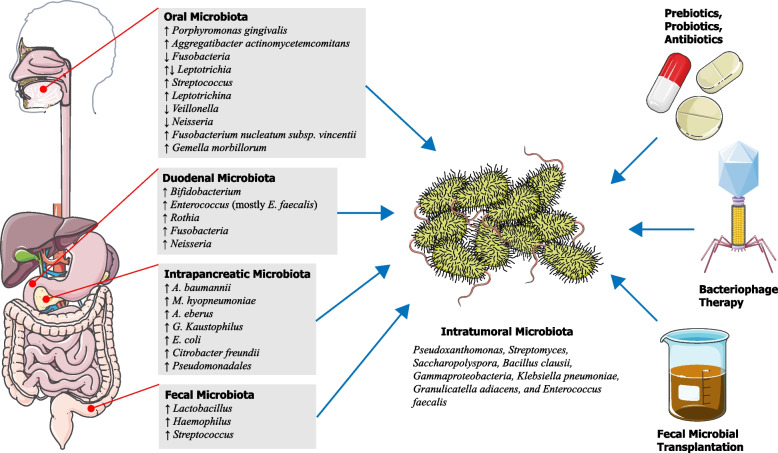
Schematic representation summarizing the role of microbiome in the development of pancreatic cancer

The intricate interplay between PC and the GI tract microbiome has been extensively investigated through numerous in vivo, in vitro, and in silico studies. Studies have been conducted on rodents in order to better understand the role of the microbiome in cancer formation (Fig. 1). In a study on mice, *P. gingivalis* was orally administered, inducing a significant change in gut microbial composition, before altering systemic inflammatory resistance [47]. Although the underlying mechanism of tumorigenesis remains uncertain, in a study by Tan et al., the presence of *P. gingivalis* was detected in both oral cavity and tumor tissues in PC patients. To prove that *P. gingivalis* can migrate from the oral cavity to pancreas, murine PC cell lines Pan02 were implanted into the pancreas of mice while gavaging *P. gingivalis*. Subsequent analysis revealed that *P. gingivalis*-gavaged mice exhibited significantly higher tumor burden and increased cell proliferation compared to vehicle-gavaged mice. Further analysis showed that *P. gingivalis* promotes PC progression by elevating the neutrophilic chemokine and neutrophil elastase secretion [48]. In recent years, there has been growing interest in understanding the role of the gut microbiome and its metabolites, particularly short-chain fatty acids (SCFAs), in PC development, progression, and clinical outcomes [49]. SCFAs, including acetate, propionate, and butyrate, are produced by the gut microbiota through fermentation of dietary fiber and other substrates [50]. SCFAs exert diverse effects on host physiology and have been implicated in various cancer types [51, 52]. Emerging evidence suggests that SCFAs can modulate several processes involved in cancer development, such as inflammation, cell proliferation, and immune responses [53]. SCFAs and the gut microbiome can influence PDAC prognosis by modulating the tumor microenvironment and host immune responses [35]. Certain SCFAs, such as butyrate, have been associated with improved prognosis and enhanced response to chemotherapy in PDAC in vitro and in vivo models [54]. Conversely, dysbiosis and alterations in SCFA production may contribute to an immunosuppressive microenvironment and poorer outcomes [35]. Interventions targeting the gut microbiota and SCFA production, such as probiotics, prebiotics, and dietary fiber supplementation, have shown potential in modulating the tumor microenvironment and enhancing immunotherapy efficacy [55, 56].

Animal models and in vitro studies have played major role in shaping our understanding of cancer formation and treatment, but clinical studies conducted on patients provide more reliable results. Fan et al. conducted a cohort study involving 361 patients with pancreatic adenocarcinoma and 371 controls matched for age, sex, race, and year of sample collection. After characterizing the composition of oral microbiota in oral wash samples using bacterial 16S rRNA sequencing, they found that *Porphyromonas gingivalis* was associated with a higher risk of PC, and the presence of *Aggregatibacter actinomycetemcomitans* was associated with a twofold increase in PDAC risk. Phylum *Fusobacteria* and the genus *Leptotrichia* were associated with a lower risk of developing PC. The possibility of reverse causation was reduced by excluding cases with neoplastic development 2 years prior to sampling. Nevertheless, the risks related to these phylotypes remained significant [57]. In order to investigate the role of *Fusobacteria* in PC development, Udayasuryan et al. found that *Fusobacterium nucleatum* infection in both normal pancreatic epithelial cells and PDAC cells caused an increase in cytokine secretion, including GM-CSF, CXCL1, IL-8, and MIP-3α, promoting phenotypes in PDAC cells associated with tumor progression, including proliferation, migration, and invasive cell motility. This phenomenon occurred in response to *Fusobacterium* infection regardless of the strain and in the absence of immune and other stromal cells. Blocking GM-CSF signaling markedly limited proliferative gains after infection [58].

In a prospective study, Wei et al. compared 41 patients diagnosed with pancreatic adenocarcinoma and 69 healthy controls [59]. 16S rRNA sequencing was used to identify bacterial taxa. Z-scores were calculated based on operational taxonomic unit values and logistic regressions were performed to calculate the risk prediction for oral bacteria. Compared to healthy controls, The study found that carriers of *Streptococcus* and *Leptotrichina* had an increased risk of PDAC development compared to healthy controls. Additionally, *Veillonella* and *Neisseria* were associated with a decreased risk of PDAC and promoted protective characteristics. Patients who reported bloating were found to be more likely to carry higher amounts of *Porphyromonas*, *Fusobacterium*, and *Alloprevotella*. Patients with jaundice had a greater abundance of *Prevotella*. Patients with dark brown urine were more abundant in *Veillonella*, and patients reporting diarrhea were found to have lower amounts of *Neisseria* and *Campylobacter*. Patients with vomiting had decreased values of *Alloprevotella*. The existence of symptoms such as bloating, jaundice, and dark brown urine might urge patients to seek medical care thus led to an earlier diagnosis and a better prognosis.

Chung et al. conducted a study in which microbiota was isolated from tongue, buccal, supragingival, and saliva samples from 52 subjects. High throughput DNA sequencing was used to characterize 16S rRNA genes. After analysis, significant difference in bacterial taxa between oral cavity and intestinal and pancreatic tissue samples were found. After adjusting for disease status and within-subject correlation, specific co-abundance patterns in the presence and absence of oral and intestinal or pancreatic samples of *Fusobacterium nucleatum* subsp. *vincentii* and *Gemella morbillorum* were observed between PC patients and healthy controls. These findings indicate that concurrent presence or absence of specific microbial clusters across different sites is associated with the progression of PC or other gastrointestinal disorders [60].

These results suggest the potential role of oral dysbiosis in the development of PDAC. Further research is required to establish any causal relationship between the oral microbiome and development of PC, as well as the possible underlying mechanism of pathogenesis. It is unknown if it is the oral microbiota affecting the composition of the intestinal or pancreatic microbiota, or the other way around. By analyzing oral samples collected from subgingival plaque, tongue coating samples, and fecal samples, Iwauchi et al. found a higher prevalence of transitions in the oral microbiota in elderly compared to other adults, suggesting the influence of oral healthcare on gut microbial composition [61]. Kohi et al. compared bacterial and fungal profiles of subjects, including 134 healthy controls, 98 pancreatic cyst patients, and 74 PDAC patients in a case-control study. PDAC patients exhibited reduced duodenal microbiota diversity compared to healthy controls and patients with pancreatic cyst, while no difference was found between the latter two groups. Significantly increased levels of *Bifidobacterium* were observed in duodenal fluid in PDAC patients compared to the healthy control group. Also, high levels of *Fusobacteria* and *Rothia* bacteria were found to be related to short-term survival (STS) of patients with PDAC. From this study, it may be concluded that the retrograde migration of pathogenic microbiota from the upper GI tract can lead to identification of diagnostic microbiome profiles of patients affected by or at risk of PDAC [62]. In another study conducted on duodenal bacteria flora of 62 patients with duodeno-pancreato-biliary cancers, 16S rRNA analyses were performed to determine bacterial composition. Of the patients, 17 were positive for *Enterococcus* spp., with *E. faecalis* showing higher survival rates in pancreatic juice compared to the other bacterial species. Thus, alkalinity may be a selective survival factor of *E. faecalis*, which is able to colonize the pancreatic duct and cause chronic changes in an altered pH condition [63]. Ren et al. divided 87 PC patients into two groups: those with pancreatic head cancer (PCH) (*n* = 54) and those with pancreatic body and tail cancer (PCB) (*n* = 31), along with 57 matched healthy controls. PCH group were further divided into two groups of obstructed bile duct (PCH-O) and unobstructed bile duct (PCH-unO). The microbial characteristics of fecal samples were analyzed using MiSeq 16S rRNA sequencing technique. The *Lactobacillus*, *Haemophilus*, and *Streptococcus* genera were found to be more abundant in stage II PC patients compared to stage I patients. Enriched *Streptococcus* was also more notable in PCH compared to PCB, and *Streptococcus* showed a significant elevation in PCH-O versus PCH-unO, marking a close association with the bile in PC. The results indicated a significant decrease in intestinal microbial diversity in PC patients, and an increase in LPS-producing pathogens were observed. Their analyses achieved high classification power for PC which can highlight the significance of gut microbiome as a potential non-invasive cancer diagnosis marker [64]. Okuda et al. detected bacteria in all isolated samples from tumor-associated tissues, gastric fluids, pancreatic juice, and bile of 11 biliary tract cancer patients and 4 PC patients who underwent curative resection. Using the detection of 16S rRNA sequences in tumor-associated tissues and pancreatic fluids, they found *Akkermansia* to have the highest abundance, but it was only detected in patients’ bile samples. They also found that patients who were positive for bile-specific *Akkermansia* were more likely to have external biliary drainage [65]. Using 16S rRNA sequencing, Riquelme et al. analyzed tumor composition in PDAC patients. They identified *Pseudoxanthomonas*, *Streptomyces*, *Saccharopolyspora*, and *Bacillus clausii* as an intra-tumoral microbiome signature in long-term survival (LTS) patients. The pancreatic microbiome, coordinated with the gut microbiome, was demonstrated to influence the host immune response and have an impact on the course of disease [44]. In a separate study by Halimi et al., pancreatic cyst fluid samples were acquired in intraductal papillary mucinous neoplastic lesions. MALDI-TOF MS profiling analysis was performed on these samples and showed *Gammaproteobacteria* and *Bacilli* dominated in isolated microbiota. Among these, *Klebsiella pneumoniae*, *Granulicatella adiacens*, and *Enterococcus faecalis* demonstrated pathogenic properties in an ex vivo culture environment. They concluded that pathogenic properties included intracellular, cell death induction, and DNA double-strand breaks, suggesting an explanation for pancreatic cystic lesions’ progression to neoplasms [66]. In a study performed by Chakladar et al., intra-pancreatic microbiome was found to be correlated with immune suppression and metastasis, in addition to a poorer prognosis of PDAC in males and smokers. They found 13 microbes in association with advanced tumor progression, while 9 were positively correlated with reduced ability of tumor suppressive pathways. Of these, *A. baumannii* and *M. hyopneumoniae* were associated with smoking, which causes genomic changes leading to PDAC. *A. ebreus*, *A. baumannii*, *G. kaustophilus*, and *E. coli* abundance were found to increase cancer activation and immune-suppression pathways in males compared to females. *Citrobacter freundii*, *Pseudomonadales* bacterium, and *A. ebreus* were positively correlated with proinflammatory immune pathway activation. *C. freundii* and *M. hyopneumoniae* were associated with immunosuppression and activation of oncogenic pathways [67]. It is worth noting that in another study conducted on the African American population, no difference in microbiota diversity between PDAC cases and healthy controls were found after accounting for multiple comparisons [68].

There is evidence to suggest that fungal and viral infections may play a role in the development of PC. The study by Aykut et al. showed that the presence of fungi, particularly *Malassezia* spp., in the pancreas is linked with the development and progression of PDAC. The composition of the mycobiome in the tumor tissue was different from that of the gut or normal pancreas. They showed that the ablation of the mycobiome could be protective against tumor growth in slowly progressive and invasive murine models of PDAC. Moreover, repopulation with *Malassezia* species accelerated oncogenesis. Further evaluation of the underlying mechanisms revealed that the ligation of mannose-binding lectin (MBL), which binds to glycans of the fungal wall to activate the complement cascade, was required for oncogenic progression [69]. The development of PC has been found to be associated with *Candida* infection in the oral cavity as per a prospective cohort study carried out in Sweden [70]. In the case of mechanism, the presence of oral *Candida* induces inflammation and promotes the growth of suppressor cells that are derived from the myeloid lineage [71]. According to certain evidence, it is possible that hepatitis viruses are connected to PC. Several studies have shown a correlation between chronic pancreatitis and hepatitis B virus [72, 73]. The risk of PC is found to be higher in individuals with hepatitis C virus infection, as proven by a meta-analysis carried out by Arafa and colleagues [74]. These investigations connect chronic hepatitis, chronic pancreatitis, and PC, highlighting the fact that the potential involvement of viruses in PC must not be disregarded.

Further study of microbial roles and underlying mechanisms and pathways is needed to gain an imperative understanding of tumor genesis associated with the microenvironment and immune pathways to encourage the development of innovative treatment and diagnostic methods. While the microbial composition of PC patients has been explored in various tissues and fluids, including pancreatic juice, bile, and tumor-associated tissues, questions about the causal relationship between the oral and intestinal microbiota and the mechanisms of pathogenesis remain. Research is required to elucidate the microbial roles, underlying mechanisms, and pathways influencing tumor genesis, microenvironment interactions, and immune responses associated with PC. Table 1 provides a summary of the microbial species discussed, delineating their respective roles in either promoting or inhibiting the progression of PC.

**Table 1:** Potential microbial species that are involved in the promotion or inhibition of progression in pancreatic cancer

Biomarker	Site	Proposed Mechanisms	Effects	Ref.
*Porphyromonas gingivalis*	Mouth	Elevation of the neutrophilic chemokine and neutrophil elastase secretion	promotes pancreatic cancer progression in vivo	[48]
*Porphyromonas gingivalis*	Mouth	initiate the Toll-like receptor signaling pathways; Evasion of the host immune system by invasion of host cells and disruption of signaling pathways by cytokine and receptor degradation;	Higher risk of pancreatic cancer	[57]
*Aggregatibacter actinomycetemcomitans*	Mouth	initiate the Toll-like receptor signalling pathways	Twofold increase in PDAC risk	[57]
Phylum *Fusobacteria* and genus *Leptotrichia*	Mouth	Immune response elicited by Leptotrichia may provide protection against pancreatic carcinogenesis	Lower risk of developing PC	[57]
*Fusobacterium nucleatum*	Intratumoral cells (In vitro)	Induced both normal pancreatic epithelial cells and PDAC cells to secrete increased amounts of the cytokines GM-CSF, CXCL1, IL-8, and MIP-3α	infection in both normal pancreatic epithelial cells and PDAC cells caused an increase in cytokine secretion, promoting phenotypes in PDAC cells associated with tumor progression	[58]
*Streptococcus* and *Leptotrichina*	Mouth	NA	Increased risk of PDAC development	[59]
*Veillonella* and *Neisseria*	Mouth	NA	Decreased risk of PDAC and promotion of protective characteristics	[59]
*Porphyromonas*, *Fusobacterium*, and *Alloprevotella*	Mouth	NA	Seen in patients reporting bloating	[59]
*Prevotella*	Mouth	NA	Greater abundance in patients with jaundice	[59]
*Veillonella*	Mouth	NA	Greater abundance in patients with dark brown urine	[59]
*Neisseria* and *Campylobacter*	Mouth	NA	Lower amounts in patients with diarrhea	[59]
*Alloprevotella*	Mouth	NA	Lowe amounts in patients with vomiting	[59]
*Fusobacterium nucleatum* subsp. *vincentii* and *Gemella morbillorum*	Oral, Intestinal, Pancreatic	Oral bacteria can pass through the oral mucosal barrier, result in abnormal local and systemic immune and metabolic responses	Specific co-abundance patterns in oral and intestinal or pancreatic samples	[60]
*Bifidobacterium*	Intestinal (Duodenal fluid)	PPIs can lead to bacterial shifts and the increase of pathogenic bacteria	Increased in PDAC patients	[62]
*Fusobacteria* and *Rothia*	Intestinal (Duodenal fluid)	PPIs can lead to bacterial shifts and the increase of pathogenic bacteria	Higher levels related with short-term survival (STS)	[62]
*Lactobacillus*, *Haemophilus*, and *Streptococcus*	Intestinal (Stool)	gut microbial functions involved in Leucine and LPS biosynthesis enriched, while Spermidine putrescine transport system and Histidine biosynthesis reduced in PC, leading to chronic inflammation	More abundant in stage II PC patients	[64]
*Streptococcus*	Intestinal (Stool)	*Streptococcus* is associated with bile acid and lipid homeostasis in the gut	More notable in PCH compared with PCB and significant elevation in PCH-O versus PCH-unO	[64]
*Akkermansia*	Bile fluid	Akkermansia is associated with the performance of external biliary drainage	More likely to be detected in patients with biliary tract cancer and external biliary drainage	[65]
*Pseudoxanthomonas*, *Streptomyces*, *Saccharopolyspora*, and *Bacillus clausii*	Tumor tissue	Tumor microbiome shapes immune responses promoting T cell activation	Intra-tumoral microbiome signature in long-term survival (LTS) patients	[44]
*Gammaproteobacteria* and *Bacilli*	Cyst fluid after resection	By inducing pancreatic cell damage including DNA repair response and cell death	Dominant in pancreatic cyst fluid in intraductal papillary mucinous neoplasm	[66]
*A. baumannii* and *M. hyopneumoniae*	Tumor tissue	part of a pathway for tobacco to influence disease severity	Associated with smoking that cause genomic changes leading to PDAC	[67]
*A. ebreus*, *A. baumannii*, *G. kaustophilus*, and *E. coli*	Tumor tissue	Increase cancer activation and immune-suppression pathways in males compared to females	Differential abundance and activation of cancer and immune-associated pathways in male versus female pancreatic adenocarcinoma patients	[67]
*Citrobacter freundii*, *Pseudomonadales* bacterium, *A. ebreus*	Tumor tissue	Proinflammatory immune pathway activation	Upregulation of oncogenic pathways	[67]
*Malassezia* spp.	Tumor tissue	Ligation of mannose-binding lectin (MBL) required for oncogenic progression	Malassezia was enriched in pancreatic ductal adenocarcinoma in both mice and humans	[69]
*Candida*	Oral	Inhabits on the mucosal epithelium and causes various oral mucosal lesions; Exerts carcinogenetic effects through production of carcinogenic byproducts, triggering inflammation, induction of the Th17 response and through molecular mimicry	Increased risk of developing pancreatic cancer in individuals with *Candida*-related oral mucosal lesions	[70, 71]
Hepatitis C virus	NA	Increased risk of diabetes and elevated pancreatic enzymes; HCV proteins interact with components of cytoplasmatic enzymes and cell cytoskeleton leading to dysregulation of transcriptional cell genome activities	Elevated risk of PC	[74]
*P. gingivalis*	Oral	TGF-β signaling pathway may be involved in the cancer-promoting effect of *P. gingivalis* and the suppressive effects of probiotics	Accelerated development of PanIN lesions in oral exposure	[138]
Pathogenic *E. coli*	Intestinal	Can activate the TUBB/Rho/ROCK signaling pathway	participates in the carcinogenesis of pancreatic cancer.	[155]

### Microbiome as diagnostic biomarker in PC

PC can influence the metabolic function of the surrounding tissue environment and potentially alter the composition of the GI microbiota, enabling researchers to identify biomarkers [75]. Current clinical practice guidelines advise primarily screening in high-risk individuals, including those with at least two first-degree relatives with PC. As a result, genetic testing must be considered for eligible relatives who are at risk for FPC [76]. Therefore, patients outside of these criteria might miss the opportunity for early intervention and treatment, which prompts the need for easily performed, non-invasive, and accurate biomarkers to broaden the screening criteria.

Oral samples are a fast way of obtaining insights into patients’ microbial composition. Although the mechanism is not yet completely clear, the literature has proven the association between PC and oral microbiota [77]. Tooth loss, cavities, and periodontal diseases have been found to be independent predictors of PC [78–80]. The oral microbiota is the most diverse environment in human body, encompassing a large variety of microbial species. Many of these oral bacteria and related dysbiosis have been found to be associated with gastrointestinal tract neoplasms. Oral bacteria can spread across various organs through blood circulation or biliary conduction, presenting their adverse effects [81].

Kim et al. used microbial extracellular vesicles (EVs) from blood samples to identify compositional differences between microbial samples of PC patients and healthy controls. The composition analysis was continued by 16 s rRNA sequencing, and prediction models were generated. At both phylum and genus levels, PC patients were found to have higher levels of *Verrucomicrobia*, *Deferribacteres*, and *Bacteroidetes* compared to healthy controls. On the other hand, *Acinetobacter* was less abundant in these patients. At the genus level only, *Lachnospiraceae, Ruminococcaceae*, *Turicibacter*, *Akkermansia, Ruminiclostridium*, and *Lachnospiraceae* were more abundant, while *Stenotrophomonas*, *Propionibacterium*, *Sphingomonas* and *Corynebacterium* were less abundant in patients with PC [82]. Baydogan et al. conducted a prospective study to investigate the oral and gut microbiome in PC patients, high-risk individuals, and healthy controls. Using 16S rRNA sequencing to analyze periodontal and stool samples, they found a significant increase in *Proteobacteria*, *Actinobacteria*, and *Fusobacteria* in the gut of PC patients, while a significant decrease in oral *Proteobacteria* was observed. The study also revealed a decreased oral/gut ratio of *Gammaproteobacteria* in PC patients compared to healthy controls, which could indicate early tumorigenesis and serve as a potential biomarker for PC. The findings suggest that detecting the Gammaproteobacteria oral/gut ratio could be an inexpensive and non-invasive method for PC screening [83]. However, another study by Lu et al. has reported an increased abundance of *Proteobacteria* in saliva, while finding no difference in tongue coating microbiome between PC patients and healthy controls [84]. In another study aimed at determining whether minimal tissue obtained by endoscopic ultrasound–guided fine-needle aspiration can be used for microbiome analysis in PC patients, Nakano et al. enrolled thirty patients who underwent the procedure 16S rRNA sequencing was performed on a total of 90 tissues, including 30 PC tissues, 30 gastric tissues, and 30 duodenal tissues. The results showed that the success rate of bacterial detection was high for all tissues, with PC tissues showing a lower bacterial diversity and a significantly different microbial structure than stomach and duodenal tissues. The study concludes that endoscopic ultrasound–guided fine-needle aspiration samples were valuable for PC microbiome analysis and revealed differences in bacterial composition compared to stomach and duodenum. They further discovered a significant increase in the relative abundance of *Proteobacteria*, *Firmicutes*, *Bacteroidetes*, and *Fusobacteria* in PC tissues compared with duodenum and stomach tissues [85]. However, results on Fusobacteria have been contradictory, as some studies have shown their lower abundance in PC [57, 86]. To detect PC in an early stage using non-invasive sample collection, Farrell et al. employed a multi-phase approach to analyze the salivary microbiota and its potential connections with pancreatic cancer and chronic pancreatitis. They employed Human Oral Microbe Identification Microarray to compare the salivary microbial composition between 10 PC patients and 10 healthy controls. They then proceeded with verification of bacterial candidates using real-time quantitative PCR, and their validation in an independent cohort of 28 PC patients, 28 healthy controls, and 27 chronic pancreatitis samples. The results revealed significant variations in the salivary microflora between patients with pancreatic cancer and healthy controls. A total of 31 bacterial species/clusters demonstrated significant increases, while 25 bacterial species/clusters showed reductions. The abundance of *N. elongata* and *S. mitis* showed significant reductions in PC patients compared to healthy controls. Conversely, the level of *G. adiacens* was significantly higher in PC patients compared to all noncancer subjects. The combination of two bacterial biomarkers, *N. elongata* and *S. mitis*, demonstrated high accuracy in distinguishing pancreatic cancer patients from healthy subjects. These findings emphasize the associations between salivary microbiota variations and PC and highlight the potential of salivary microbiota as a source for non-invasive biomarkers in systemic diseases [87]. On the other hand, Torres et al. found no difference in *S. mitis* levels between saliva samples of PC patients and healthy individuals [88]. In a recent study by Yang et al., 16S rRNA sequencing on DNA extracted from fecal samples from 44 PC patients and 50 healthy controls were performed to compare gut microbiota profiles. Using LEfSe, random forest modeling, and ROC curve analysis, they identified *Streptococcus* as a potential non-invasive biomarker that was more abundant in PC patients, especially in those with liver metastasis. Their results suggest that *Streptococcus* abundance could be a useful screening tool for early detection of PC [89]. Another study demonstrated that individuals with an antibody level equal to or exceeding 200 ng/ml against *Porphyromonas gingivalis* exhibit a twofold higher risk of developing pancreatic cancer compared to those with antibody levels below 200 ng/ml. Therefore, the presence of periodontal diseases may serve as a potential risk factor for pancreatic cancer. Notably, their results suggested that an elevation in antibody levels against distinct types of oral bacteria could be beneficial in terms of cancer prevention [90]. Researchers conducted a study to explore fecal and salivary microbiota as potential diagnostic biomarkers for PDAC. They applied shotgun metagenomic and 16 s rRNA sequencing to Spanish and German populations, including 57 cases and 50 controls. The study found that the fecal metagenomic classifiers outperformed the saliva-based classifiers in identifying PDAC. The classifier identified patients with PDAC with an accuracy of up to 0.84 area under the receiver operating characteristic curve (AUROC) based on a set of 27 microbial species, with consistent accuracy across early and late disease stages. The research also found that the performance further improved to up to 0.94 AUROC when combining the microbiome-based predictions with serum levels of CA19–9. Moreover, the classifier was validated in an independent German PDAC cohort and confirmed against 25 publicly available metagenomic study populations with various health conditions. The PDAC-specific microbiome signatures identified could offer new microbiome-related hypotheses regarding disease etiology, prevention, and possible therapeutic intervention [30]. Although recent research has been promising, many challenges persist in identifying reliable biomarkers. Half et al. found 14 bacteria using 16 s rRNA method, which could distinguish between PC and healthy control groups. Despite these results, high inter-subject variability was noted, and only a small portion of PC-related bacterial signals were found in patients with pre-cancerous lesions [91]. Additionally, some of the identified bacteria proposed as diagnostic biomarkers are not consistently present in all PC patients. Possible factors contributing to the observed variabilities in PC patients include age, ethnicity, lifestyle, geographic location, dietary intake, and gender [92, 93]. Moreover, alcohol consumption and bowel movement quality have been observed to influence gut microbiome variance in affected patients compared to healthy controls. Li et al. analyzed the saliva microbiome from native Alaskans, Germans, and Africans and found that there were significant differences in the diversity between and within individuals across the three groups. The results indicated that there were more similarities in the saliva microbiome of Native Alaskans and Germans than between either group or Africans. The study also highlighted the distinctiveness of the saliva microbiome of human groups living under very different climatic conditions [93]. Another study compared the microbiota of infants from Southeastern Africa and Northern Europe. Malawian infants had higher levels of *Bifidobacterium*, *Bacteroides-Prevotella*, and *C Histolyticum* compared to Finnish infants, which was most likely due to their diet high in plant polysaccharides introduced through breast-feeding. Malawian infants also had a higher *Firmicutes/Bacteroidetes* ratio, which has been associated with obesity in previous studies [94]. Regarding the pancreatic cancer-associated microbiome in different geographical areas, several studies have reported varying results. For instance, Maisonneuve and Lowenfels conducted a meta-analysis, which concluded that the presence of *H. pylori* could be linked to 4 to 25% of PC cases in Western countries [95]. A study conducted by Wang et al. highlighted the geographical variations in the correlation between *H. pylori* and PC. The study indicated that individuals with CagA+ are more prone to developing the disease in western countries rather than in eastern countries [96]. Furthermore, a comprehensive examination of regional data regarding the role of hepatitis B virus in the development of PC revealed that the virus had a substantial impact only in Asia and Oceania, while it was inconsequential in Europe [97].

These variabilities arising from lifestyle and physiological differences lead to false positives and limit our understanding of the relationships between microbiota composition and pathologies [92, 98]. An inherent challenge encountered when utilizing gut microbiota as diagnostic markers in research studies is the substantial variability and fluctuation in microbial composition observed among individuals. This dynamic nature of the gut microbiota poses a potential obstacle to accurately identifying biomarkers and developing targeted therapeutic approaches [99]. The diverse dietary habits of individuals also significantly influence microbial composition and stability, thereby contributing to the complexity involved in designing these studies [100]. In a study by del Castillo et al., 16S rRNA sequencing was conducted on 189 pancreatic and duodenal samples of 77 subjects. They found that the bacterial DNA in the pancreas and duodenum were highly subject-specific in both cancer patients and non-cancer subjects, which adds to the complexity of this matter [101].

Despite all the mentioned challenges, the use of microbiome analysis as a diagnostic marker holds several potential advantages and practical implications in clinical settings. Employment of easily-performed, accessible, non-invasive, and accurate biomarkers can broaden the current screening criteria that is mostly recommended for high-risk individuals based on genetics. Analyzing microbial extracellular vesicles from blood samples and conducting 16S rRNA sequencing on fecal and saliva samples have identified potential bacterial biomarkers for PC. Furthermore, combining microbiome findings with serum levels of CA19–9 further improves diagnostic accuracy. Overall, these results suggest that using microbial composition and diversity across PC patients as diagnostic biomarkers is an achievable yet challenging goal. It should be noted that there remains contradiction between results of different studies regarding differences in microbial composition. As such, further studies are necessary to strengthen the diagnostic role of these novel biomarkers in both clinical and preclinical settings. Table 2 summarizes the discussed microbial species that can serve as diagnostic biomarkers in PC.

**Table 2:** Potential diagnostic biomarkers of pancreatic cancer

Biomarker	Site	Proposed Mechanisms	Effects	Ref.
phylum and genus *Verrucomicrobia*, *Deferribacteres*, and *Bacteroidetes*	Blood microbial extracellular vesicles	*Akkermansia* (in *Verrucomicrobia* phylum) is an immune modulator related to the programmed cell death protein 1 blockade pathway	Higher in PC patients compared to HC	[82]
Genus *Lachnospiraceae, Ruminococcaceae*, *Turicibacter*, *Akkermansia, Ruminiclostridium*	Blood microbial extracellular vesicles	*Akkermansia* is an immune modulator related to the programmed cell death protein 1 blockade pathway	Higher in PC patients compared to HC	[82]
phyllum and genus *Acintobacter*	Blood microbial extracellular vesicles	*Actinobacteria* are known to produce butyrate and modulate immune function	Less abundant in PC patients compared to HC	[82]
Genus *Stenotrophomonas*, *Propionibacterium*, *Sphingomonas* and *Corynebacterium*	Blood microbial extracellular vesicles	May lead to an increased amount of acute phase inflammatory cytokines which might pave the way for cancer	Less abundant in PC patients compared to HC	[82]
*Proteobacteria, Actinobacteria*, and *Fusobacteria*	Oral cavity and gut	NA	Significant increase in Proteobacteria, Actinobacteria, and Fusobacteria in the gut of PC patients, while a significant decrease in oral Proteobacteria was observed.	[83]
*Gammaproteobacteria*	Oral cavity and gut	NA	Decreased oral/gut ratio in PC patients compared to healthy controls, which is indicative of early tumorigenesis	[83]
*Proteobacteria*	Pancreas tissue	NA	Significant increase in PC tissues compared to duodenum and stomach tissues	[85]
*Firmicutes, Bacteroidetes, and Fusobacteria*	Pancreas tissue	NA	Significant decrease in PC tissues compared to duodenum and stomach tissues	[85]
*N. elongata and S. mitis*	Oral cavity	*S. mitis* plays a protective role against the adhesion of cariogenic bacteria	Significant reductions in PC patients	[87]
*G. adiacens*	Oral cavity	associated with systemic inflammations	Significantly higher in PC patients	[87]
*S. mitis*	Oral cavity	The proliferation of periodontal pathogens leads to systemic inflammation and cancer progression	No difference in levels between saliva samples of PC patients and healthy individuals	[88]
*Streptococcus*	Gut	NA	More abundant in PC patients, especially in liver metastasis	[89]
*Porphyromonas gingivalis*	Oral cavity/Plasma (antibodies)	elevated levels of antibodies to oral bacteria serve as a marker for a genetically stronger immune response, providing protection against carcinogenesis	Elevated anti-*Porphyromonas gingivalis* antibody beneficial for cancer prevention	[90]
Hepatitis B virus	Liver	Chronic inflammation leading to malignant transformation, HBV DNA integration disrupting tumor suppressing gens	Impact on PC development in Asia and Oceania, but inconsequential in Europe	[97]
*Streptococcus* and *Veillonella*	Gut	Induce interleukin (IL)-6, IL-8, IL-10, and tumor necrosis factor-a reactions in dendritic cells	Abundance in PDAC patients	[36]
*Faecalibacterium prausnitzii*	Gut	Short-chain fatty acids regulate intestinal immune functions through cell surface G-protein coupled receptors, and their decrease leads to inflammation	Reduced numbers in PDAC patients	[36]
*Faecalibacterium, Parvimonas, Alistipes*, and *Anaerostipes*	Gut	Less suppression of proinflammatory and promotion of anti-inflammatory cytokines	Decrease in PDAC patients	[109]
*Anaerotruncus, Pseudonocardia, Mucispirillum,* and *Cloacibacterium*	Gut	NA	Increased PDAC patients	[109]

### Microbiome as prognostic biomarker in PC

There is a need to develop new clinical approaches and personalized treatments to manage PC. It is important to identify potential microbial profiles as tools to indicate the progression of the disease and help clinicians with prognostic outcomes in PC patients [102]. Weniger and colleagues preformed a retrospective study on 211 patients with available biliary fluid cultures. The data revealed an association between a greater number of pathogen species detected in bile cultures and a decline in progression-free survival (− 1.9 (95% Confidence Interval − 3.3 to − 0.5) months per species; *P* = 0.009). The use of gemcitabine as an adjuvant treatment improved progression-free survival in patients who tested negative for *K. pneumoniae* (26.2 versus 15.3 months; *P* = 0.039), but not in patients who tested positive (19.5 versus 13.2 months; *P* = 0.137). The use of Quinolone was associated with improvements in the median overall survival of patients, regardless of *K. pneumoniae* results (48.8 versus 26.2 months; *P* = 0.006) and for patients with positive *K. pneumoniae* tests (median not reached versus 18.8 months; *P* = 0.028). The presence of quinolone-resistant *K. pneumoniae* in patients led to a shorter progression-free survival compared to those with quinolone-sensitive *K. pneumoniae (9.1* versus *18.8 months; P = 0.001)* [103]. In a cohort study performed by Kirishima and associates on 244 patients, microbiome-derived DNA was extracted and studied from bile juice in surgically extracted gallbladders. Kirishima et al. found no significant difference in the microbiome composition based on lesion position and cancer type in regard to alpha and beta diversity. *Enterobacter, Hungatella, Mycolicibacterium, Phyllobacterium* and *Sphingomonas* revealed a significant difference in PDAC, between stages with and without lymph node metastasis. The use of Cox proportional hazards model revealed that a high relative abundance of *Enterococcus, Eggerthella, Klebsiella, Corynebacterium, Moraxella, Hungatella, Paracoccus, Dermacoccus, Citrobacter, Lawsonella* and *Pseudoxanthomonas* led to a significant worsening of patients’ prognoses (Hazard ratio (HR) = 1.65, 2.22, 2.21, 2.36, 2.27, 2.74, 2.50, 3.14, 2.60, 3.48 and 7.41, respectively). Patients with *Streptococcus, Escherichia, Veillonella,* and *Dialister* in relatively higher amounts experienced significantly better prognoses (HR = 0.60, 0.59, 0.50, and 0.35, respectively) [104]. To investigate the potential role of *Fusobacterium* in PC, Mitsuhashi and colleagues examined 283 PDAC patients for the presence of *Fusobacterium* species within their cancerous tissue samples. They successfully identified *Fusobacterium* species within 8.8% of the PC tissue samples, when comparing the median cancer-specific survival between the two groups, the *Fusobacterium* species-positive group had significantly lower shorter survival (17.2 months versus 32.5; log-rank *P* = 0.021) and came to the conclusion that the presence of *Fusobacterium* species is independently associated with a more unfavorable prognosis, indicating *Fusobacterium* species may serve as another prognostic biomarker [105]. In a multinational study conducted by Nagata et al., salivary and fecal samples were collected from both treatment naïve PDAC patients and non-PDAC controls, and metagenomic classification was applied. The results indicated an association between oral and gut dysbiosis and PDAC, along with identifying a significant abundance of *Streptococcus* and *Veillonella*, in contrast to reduced levels of *Faecalibacterium prausnitzii* as being gut signatures associated with PDAC*.* Furthermore, higher abundance of *F. prausnitzii* (HR = 0.5), *Alistpies* (HR = 0.4), and *Enterobacteriaceae* (HR = 0.6) species in the gut microbiome and *Capnocytophaga* (HR = 0.6) in the oral microbiota were indicative of lower mortality rates. Conversely, *R. torques* (HR = 1.3) in the gut, and *S. vestibularis* (HR = 1.9) and *N. bacilliformis* (HR = 1.4) in the oral cavity were associated with poorer prognosis [36]. In another study, Murthy et al. investigated the prognostic role of the gut microbiome in patients with PDAC undergoing neoadjuvant therapy (NAT) prior to surgery. The researchers collected fecal samples from 42 patients with localized PDAC and performed 16S rRNA sequencing on fecal samples obtained before NAT as well as resected tumor samples to assess the influence of the baseline gut microbiome on clinical outcomes. The study found that the gut microbiota of NAT responders had an increased proportion of *Akkermansia*, which activates the adaptive immune system, while NAT non-responders had an increased proportion of *Enterobacteriaceae*, which metabolizes gemcitabine (Additive log ratio (ALR) -3.4 ± 2.0 vs − 8.7 ± 0.8, *P* = 0.0004). Interestingly, the study also found that the tumor microbiota exhibited reduced diversity compared to the gut microbiota and was not associated with clinical outcomes. Additionally, incorporating gut microbiota data enhanced the predictive ability of the model for NAT response and survival. These findings suggest that the gut microbiota could serve as a potential biomarker for NAT response and prognosis in PDAC patients and warrant further investigation [106]. Cancer metabolic phenotypes have been extensively investigated by metabolic analyses, allowing for the identification of diagnostic and prognostic biomarkers to improve therapeutic management [107, 108]. Guo et al. discovered a decrease in gut *Faecalibacterium, Parvimonas, Alistipes*, and *Anaerostipes* in patients with PDAC. Meanwhile, gut *Anaerotruncus, Pseudonocardia, Mucispirillum,* and *Cloacibacterium* were found to be more abundant. They also identified *Coprococcus catus*, *colesterdium hathewayi*, genera *Alistipes* and *Anaerostipes* as protective factors which were positively correlated with patient survival time. Metabolomics analyses proved significant alterations of amino acids, lipids, fatty acids, and carnitine derivatives. Additionally, disruption in signaling pathways and mitochondrial dysfunction were associated with changes in these metabolites, providing insight into the survival time of PDAC patients [109]. Regarding nonbacterial biomarkers, according to the findings of Wei et al., the presence of hepatitis B virus infection in individuals with PDAC resulted in a higher occurrence of simultaneous liver metastasis compared to the individuals classified as HBsAg negative (46.0% vs 32.0%, *P* = 0.03). This condition was identified as an independent prognostic marker [110]. Table 3 summarizes our current knowledge on the microbiome as a prognostic marker of PC. It can be suggested that microbial composition and diversity in different parts of the GI tract may potentially offer insight into survivability and prognosis in PC patients. The association between specific microbial signatures and patients’ prognoses and its potential indication of possible treatment strategies emphasizes the relevance of microbial composition in PC progression. However, further studies are required to better illustrate the association between these components.

**Table 3:** Potential prognostic biomarkers of pancreatic cancer

Biomarker	Site	Proposed Mechanisms	Effects	Ref.
*K. pneumoniae*	Biliary fluid	Gemcitabine metabolism and resistance plus other possible yet undiscovered effects	If negative, improved progression-free survival	[103]
Quinolone-resistant *K. pneumoniae*	Biliary fluid	Quinolones induce long-lasting alterations in the microbiome leading to changes in tumor biology	Shorter progression-free survival	[103]
*Campylobacter*, *Citrobacter* and *Leptotrichia*	Gallbladder	NA	Increased in cholangiocarcinoma	[104]
*Enterobacter, Hungatella, Mycolicibacterium, Phyllobacterium* and *Sphingomonas*	Gallbladder	NA	Significant difference in pancreatic ductal adenocarcinoma, between stages with and without lymph node metastasis	[104]
*Enterococcus, Eggerthella, Klebsiella, Corynebacterium, Moraxella, Hungatella, Paracoccus, Dermacoccus, Citrobacter, Lawsonella* and *Pseudoxanthomonas*	Gallbladder	NA	Higher amounts lead to worse prognoses	[104]
*Streptococcus, Escherichia, Veillonella* and *Dialister*	Gallbladders	NA	Patients with higher amounts have better prognoses	[104]
*Fusobacterium*	Pancreas	Lead to inflammation, reactive oxygen species, and epigenetic changes	Worse prognoses	[105]
*F. prausnitzii, Alistipes,* and *Enterobacteriaceae* species	Gut	Survival analysis revealed associations of favorable prognosis with higher abundances of short-chain fatty acid producers in the gut	Abundance in gut microbiome indicator of lower mortality	[36]
*Capnocytophaga*	Oral cavity	NA	Abundance in oral microbiome indicator of lower mortality	[36]
*R. torques*	Gut	linked with inflammatory bowel disease	Poorer prognosis	[36]
*S. vestibularis*, and *N. bacilliformis*	Oral Cavity	NA	Poorer prognosis	[36]
*Akkermansia*	Gut	Adaptive immune system activation	increased proportion in the gut microbiota of NAT responders	[106]
*Enterobacteriaceae*	Gut	Gemcitabine metabolization	increased proportion in the gut microbiota of NAT non-responders	[106]
*Coprococcus catus*, *colesterdium hathewayi*, genera *Alistipes* and *Anaerostipes*	Gut	A positive correlation exists between the abundance pf *Alistipes* and saturated long-chain fatty acids that are decreased in unresectable PDAC compared to resectable PDAC	positively correlated with survival time	[109]
*Alistipes, Anaerostipes, Faecalibacterium and Parvimonas*	Gut	Abundance of *Faecalibacterium* was reduced in unresectable PDAC and positively correlated with phosphatidylcholine, an indicator of less serious physiological state;	reduction in unresectable PDAC patients	[109]
*Pseudonocardia, Cloacibacterium, Mucispirillum, and Anaerotruncus*	Gut	Anaerotruncus is associated with LysoPE that is decreased in unresectable PDAC	Increase in unresectable PDAC patients	[109]
Hepatitis B virus	Liver	Inflammation and down regulation of anti-cancer cytokines	Higher occurrence of simultaneous liver metastasis in PDAC	[110]
*Gammaproteobacteria*	Intratumoral	Metabolizes and deactivates gemcitabine	Metabolize and deactivate gemcitabine leading to drug resistance	[37]

### Microbiome as potential therapeutic target

Currently, the primary method of PC treatment is surgery, chemotherapy, and radiation therapy. Although these treatments can provide some benefits, they often have limited success and can cause significant side effects. Novel methods such as personalized bacteriophage therapy, immune modulation, and fecal microbial transplantation may offer promising alternatives to traditional treatments for PC. Recent studies have shown that the modulation of microbial populations in the GI tract and pancreas is a potential strategy for PC prevention and treatment (see Table 4). Finding particular species that are particularly abundant in certain tissues and are increased in GI neoplasms can aid in the development of potential therapeutic targets [111–113]. Better outcomes have been recorded in the treatment of a variety of malignancies using immunotherapy [114, 115], fecal microbial transplantation [116], prebiotics and probiotics [117, 118], and antibiotics [119, 120] that lead to changes in microbial diversity, thus affecting treatment or progression of dysbiosis. Despite the lack of extensive research on PC, microbiome modulation has been explored as a potential alternative therapy for treating PC.

**Table 4:** Therapeutic strategies to target microbiota in pancreatic cancer

Agent	Effects	Ref.
bacteriophage therapy	Can be used to target and control specific bacteria such as *Klebsiella, Fusobacterium* and *Acinetobacter baumannii*	[126, 127]
ferrichrome	Produced from *Lactobacillus casei* and can inhibit the growth of pancreatic cancer cells. Altered the expression of p53-associated mRNAs.	[138]
caerulein	Reduced Smad3 and phosphorylated Smad3 expression in mice. Reducing the proliferation and viability of cancer cells, inhibiting PanIN progression, and metastasis. Improved patient’s tolerance of chemotherapy. Inhibitory effect on PanIN changes and serum liver enzyme elevation.	[54, 139]
butyrate	proliferation and enhancing gemcitabine effectiveness. Reduced cancer-associated stromatogenesis, preserved intestinal mucosa integrity, affected fecal microbiota composition, and ameliorated some markers of kidney and liver damage	[140]
*Megasphaera*	Improved anti-PD-1 treatment	[141]
Resistant starches	influence microbial community, increase short chain fatty acids synthesis, and protects against DNA damage	[54, 140]
UA in conjunction with GEM	Suppression of the RAGE/NF-κB/MDR1 cascade and restriction of the growth of subcutaneous tumors in mice	[155]
Ciprofloxacin	*Removes Gammaproteobacteria* which can metabolize and deactivate gemcitabine	[152]
Gemcitabine	Decreased the proportion of *Firmicutes* and *Bacteroidetes* Increased *Proteobacteria*, *Verrucomicrobia*, with a rise in bacteria associated with inflammation in mice	[157]
Vancomycin, Neomycin, Metronidazole, Ampicillin and Amphotericin B	increase in interferon gamma-producing T cells, and a decrease in interleukin 17A and interleukin 10-producing T cells	[158]

### Bacteriophage therapy

The administration of bacteriophage therapy has been useful in combating bacterial biofilms [121] and controlling bacterial infections, as a complementary treatment to antibiotics [122]. Bacteriophages are viruses that infect and eliminate their bacterial host [123]. Recent advances have enabled phage therapy as an ideal compassionate treatment due to the lack of adverse effects and its usage in the treatment of drug-resistant bacterial infections [124, 125]. In a systematic review, Kabwe et al. identified and categorized bacteria found in abundance along the IG tract in four locations. Furthermore, they highlighted bacteria present in more than one site. Of these bacteria, *Klebsiella* and *Fusobacterium* have well documented phages that could be utilized as a novel treatment for PC [126]. In a case study of a diabetic 68-year-old patient, personalized bacteriophage therapy was administered to successfully treat pancreatitis caused by *Acinetobacter baumannii.* In this patient, the treatment using antibiotics had led to deterioration in a course of 4 months. The infection was cleared using 9 different bacteriophages personalized for the patient by identifying the *Acinetobacter baumannii* isolate lytic activity in the patient [127]. As previously discussed, *Acinetobacter baumannii* can activate pathways that contribute to the development of PC, and thus, bacteriophage therapy may hold potential as a therapeutic approach for PC treatment and prophylaxis. Chronic pancreatitis is a known risk factor for the development of PC [128, 129]. This successful treatment can be indicative of the usefulness of phages in combating chronic pancreatic infections and potentially preventing the development of PC. The potential employment of phages to modulate the immune system presents a promising avenue for treating PC [130, 131]. Phages are able to alter immunity in bacterial infections by recruiting phagocytosis and inducing cytokine responses in the innate immune system, as well as antibody production in the adaptive immune system [132]. Phages are also able to cure antibiotic-resistant infections effectively in immunocompromised cancer patients with solid tumors or hematological malignancies, in addition to improving the immune response [133].

### Probiotics

Probiotics are defined as “living microorganisms that, when administered in adequate amounts, confer health benefits on the host” [134]. Dietary probiotics are commonly available as functional foods or supplements. The ability of probiotics to modulate signaling pathways, including MAPK and NF-κB, provides benefits to consumers through immunomodulation and manipulation of intestinal microbiota composition [135, 136].

Kita and colleagues have found that ferrichrome, derived from *Lactobacillus casei*, probiotic bacteria, inhibits the growth of PC cells, even those resistant to 5-FU as shown by in vitro and in vivo testing in a mouse xenograft model. Ferrichrome dysregulated the cell cycle by activating p53, thereby preventing the progression of cancer cells. Ferrichrome may also lead to apoptosis in PC cells as evidenced by DNA fragmentation and cleavage of poly (ADP-ribose) polymerase following ferrichrome treatment. Ferrichrome also significantly altered the expression of p53-associated mRNAs. The tumor-suppressive effects of the probiotic-derived ferrichrome in colorectal and gastric cancer has also been demonstrated by Kita et al. Probiotic-derived molecules, such as ferrichrome, may be the reason behind the tumor-suppressive effects of probiotics, making these molecules potential candidates for use as antitumor drugs, even in refractory and 5-FU-resistant PC [137]. In a study conducted on mice aimed at analyzing the role of probiotics and *Porphyromonas gingivalis*, Chen and associates found that the weight of pancreas in mice treated with *P. gingivalis* accompanied by probiotics was less than that of mice treated solely with *P. gingivalis*, and in terms of gene expression, genes associated with tumor growth were noticeably less expressed in the pancreas. The use of probiotic also led to reduced Smad3 and phosphorylated Smad3 expression in KC mice treated with *P. gingivalis*. Chen et al. showed that the development of PanIN lesions could be accelerated by oral exposure to *P. gingivalis,* and that probiotics may prove beneficial by reducing the proliferation and viability of cancer cells, inhibiting PanIN progression, and metastasis. The tumor suppressive effects observed in probiotics might be explained by their involvement in the transforming growth factor-β signaling pathway [138]. In a different study conducted by Chen and colleagues on mice, they found that the use of gemcitabine and probiotics led to a milder grade of pancreatic intraepithelial neoplasia formation, a reduction in vimentin and Ki-67 expression, as well as lower serum liver enzymes (AST, ALT). High-dose probiotics were also tested independently, which inhibited PanIN changes and the elevation of serum liver enzyme. The data analyzed by Chen et al. suggest that the effectiveness of chemotherapy and the patient’s tolerance of chemotherapy can be improved with the use of probiotics [139]. In another study, Panebianco et al. investigated the efficacy of butyrate, which is produced as a result of bacterial fermentation of dietary fibers, in slowing down the proliferation and enhancing gemcitabine effectiveness against two human PC cell lines in vitro. In a mouse model of PDAC, butyrate markedly reduced cancer-associated stromatogenesis, preserved the intestinal mucosa integrity, affected fecal microbiota composition, and ameliorated some markers of kidney and liver damage. These findings suggest that butyrate supplementation may interfere with pancreatic cancer biology and its response to treatment, and mitigate the damage associated with cancer or chemotherapy, alongside conventional therapies [54]. In another study by Huang et al., tumor samples were collected from PDAC survivors and analyzed using 16S rRNA gene sequencing. In addition to significant associations between microbial composition and survival time, *Megasphaera* administration in combination with anti-PD-1 treatment inhibited tumor growth in mice. These results support the possible interaction between various microorganisms and anti-tumor mechanisms in PC patients [140]. As these studies have shown, probiotics exhibit several significant therapeutic effects in different stages of PC treatment. However, the underlying mechanisms are yet to be fully revealed. These finding warrant deeper examination as no clinical trials have been conducted to this date.

### Prebiotics

Prebiotics are beneficial nutrients for the host that are utilized by the GI microbiota and manipulate the intestinal microenvironment [141]. There are many dietary strategies employed to modulate the microbial composition by consumption of fiber-rich food or prebiotics, defined as selectively fermented ingredients that cause alterations in the composition and activity of the GI microbiota, leading to benefits for the host [142, 143]. Resistant starches are among dietary fibers considered as prebiotics, which influence microbial community, increase SCFA synthesis, and protect against DNA damage [144–146]. In an investigation of the association between resistant starch (RS) and PC in xenograft mice, RNA-Seq in tumor tissue exhibited alterations in gene expression of the carbohydrate metabolism network. The serum samples were evaluated, and several compounds including xanthine, hypoxanthine, and inosine were decreased in the RS group compared with the controls [147]. These compounds are related to purine metabolism and the salvage pathway. Higher levels of these compounds have been found to be correlated with cancer and are commonly found in tumor cells [148]. In a similar study conducted on xenograft mice, miRNA expression profiles exhibited 19 dysregulated miRNAs in the RS group compared with controls. The upregulation of *miRNA-375, miRNA-148a-3p, miRNA-125a-5p*, and *miRNA-200a-3p* in the RS group was correlated with a better prognosis of PC. It was found that these miRNAs involved in RS digestion were related to genes that regulate tumor growth and metastasis [149]. The mechanism of action of prebiotics is suggested to be related to anti-adhesiveness against pathogens. Prebiotics achieve this trait by interacting with bacterial receptors through the imitation the microvillus glycoconjugates, thus avoiding the attachment of pathogens to epithelial cells [150, 151]. The wide range of uses for prebiotics is well documented in a few cancers. That being said, when it comes to pancreatic cancer, more research is required to better understand their applications as possible treatment options in clinical settings.

### Antibiotics

Antibiotics are known to alter tumor sensitivity to many drugs and therapies, leading to an elevated risk of cancer in susceptible patients. On the other hand, some studies have found that the use of antibiotics as therapeutic agents has been beneficial in the treatment of PC, due to the inhibition of bacteria with the ability to metabolize gemcitabine into an inactive form [37, 152, 153]. In order to study the role of ursolic acid (UA) in dealing with cancer drug resistance, Li and collaborators conducted cell culture and mouse studies. They found that advanced glycation end products (RAGE), pP65, and multidrug resistance protein 1 (MDR1) protein expression can be downregulated with UA treatment, and with RAGE siRNA in PC cells resistant to GEM after transfection. The use of UA in conjunction with GEM, suppressed the RAGE/NF-κB/MDR1 cascade and restricted the growth of subcutaneous tumors. GEM-treated mice experienced decreased α-diversity of gut microbiota with a significant decrease in ratio of Firmicutes/Bacteroidetes, but UA or UA plus gemcitabine treatment caused a slight increase in their proportion. The group treated with GEM had lower relative abundance of *Anaeroplasma*, the *Eubacterium xylanophilum* group, and *Roseburia,* but higher levels of *Parabacteroides* and *Ruminiclostridium 6* in comparison to the control group. However, UA treatment repressed the relative abundance of *Ruminiclostridium 6* to lower than control group. The relative abundance of *Erysipelatoclostridium* saw a significant increase, whereas decreases in *Mucispirillum* and *Ruminiclostridium 6* were observed in mice who were treated with UA plus GEM compared to those treated with GEM only. The data suggests that UA administration may directly influence the relative abundance of *Ruminiclostridium 6* in mice [154]. Geller and colleagues conducted a study on colon cancer models and found that the bacterial enzyme cytidine deaminase in its long isoform (CDD_L_), mainly observed in *Gammaproteobacteria*, can metabolize and deactivate gemcitabine leading to drug resistance of the tumor. They found that the use of the antibiotic ciprofloxacin can mitigate this drug resistance aspect. Despite having conducted the study on colon cancer, they also hypothesized that intratumor bacteria can play a part in the drug resistance of PDAC. Supporting this claim, they found that out of 113 human PDAC they tested, 76% were positive for bacteria, mainly *Gammaproteobacteria* [37]. The study done by Luo and colleagues identified the gene *TUBB* (tubulin, beta class I) as having an association with pathogenic *E. coli* infection, which can activate the TUBB/Rho/ROCK signaling pathway and might thus participate in the development and carcinogenesis of PC. They propose a novel PC therapy target in the form of co-treatment with antibiotics alongside TUBB/Rho/ROCK signaling inhibitors [155]. In mice with PC studied by Panebianco and associates, the use of gemcitabine noticeably decreased the proportion of *Firmicutes* and *Bacteroidetes* while leading to an overall increase in *Proteobacteria*, including *E. coli* and *Aeromonas hydrophila*, and also in *Verrucomicrobia*, including *Akkermansia muciniphila*, with a rise in bacteria associated with inflammation [156]. A study done by Sethi and associates investigating the modulatory effects of gut microbiota on the immune response found that the depletion of gut microbiota with the use of oral antibiotics can significantly reduce tumor burden. But this reduction in tumor growth did not hold true for Rag1-knockout mice, which lack mature T and B cells. With the use of flow cytometry, it was determined that the depletion of gut microbiota caused a significant increase in interferon gamma-producing T cells and a decrease in interleukin 17a and interleukin 10-producing T cells [157].

Further studies have been conducted to investigate the effects of neoadjuvant therapy on the biliary microbiome. One study performed by Goel and colleagues found that the bile of patients who had received neoadjuvant therapy prior to surgery possessed a higher likelihood of containing *enterococci* and *Klebsiella* compared to the bile of patients who underwent surgery without neoadjuvant therapy. Neoadjuvant therapy also made cephalosporin resistance more common in patients [158]. A similar study carried out by Nadeem and colleagues found a significant increase in the growth of gram-negative anaerobic bacteria in patients who had received neoadjuvant therapy. Nadeem et al. also found more strains with resistance to ampicillin-sulbactam, cefazolin, cefoxitin, and cefuroxime in patients who did not undergo neoadjuvant therapy [159]. Both studies found the incidence of SSIs was not affected by neoadjuvant therapy, and both suggested perioperative antibiotic prophylaxis for gram-negative bacteria and *enterococci*.

A multitude of studies have shown that the use of antibiotics negatively affects the outcome of cancer treatment. As mentioned, antibiotics can alter the microbial diversity and abundance in the GI tract, causing changes in immune responses and inflammation. In a healthy state, the intestinal microbiota stimulates specific immune cells, enhancing antibody production and strengthening immunity. Imbalance in the microbiota can impair immune function, leading to a systemic inflammatory response that may contribute to cancer development [160]. A review of the literature seems to indicate that the use of broad-spectrum antibiotics can lead to poorer clinical outcome in various cancer patients treated with immune checkpoint inhibitors, that is recommended for the rare subset of PC patients with deficient mismatch repair or high tumor mutational burden [161]. Additionally, while neoadjuvant therapy is widely researched, there is still limited data to support this approach [162]. Moreover, the overt use of antibiotics in high concentrations might lead to the emergence of resistance in intracellular bacteria such as *Porphyromonas gingivalis*, which is involved in tumorigenesis of PC [163–165]. Additionally, antibiotic resistance has been implicated in the loss of treatment efficacy in cancer patients [166]. Finding means to manage and prevent antibiotic-resistant infections which may lead to chronic infections and subsequent cancer is a major topic in need of more thorough investigation.

### Fecal microbiota transplantation

Fecal microbial transplantation has demonstrated significant efficacy against many GI pathogens which helps with modulating and treatment of dysbiosis and associated cancers [167]. In order to assess the relationship between the gut microbiome and metabolic and immune-related variables, Genton and associates performed fecal material transplantation (FMT) from PC patients and healthy controls into gram-free mice. They found that mice from both groups had similar gains in body weight and food intake, but visceral fat was lower in mice with FMT from PC patients. There was no significant difference in all other non-metataxonomic parameters among the two groups. *Clostridium scindens*, *Clostridium bolteae,* and *Phascolarctobacterium faecium* were found in higher proportions among patients with PC and in mice transplanted with the feces from these patients. In contrast, lower amounts of *Alistipes obesi*, *Coriobacteriaceae* and *Lachnospiraceae* species were observed [168]. Comparing the tumor microbiota of PDAC patients based on the short-term (STS) and long-term survival (LTS) of the patients, Riquelme and collaborators found that LTS patients had a higher alpha-diversity. Furthermore, an intra-tumoral microbiome signature was identified in LTS patients, including *Pseudoxanthomonas, Streptomyces, Saccharopolyspora, Bacillus clausii*. This signature was indicative of long-term survivorship and thus, considered a strong prognostic indicator. To investigate the role of the microbiome in PDAC, Riquelme et al. conducted FMT experiments in mice using samples obtained from LTS, STS, and healthy control donors. Riquelme et al. observed that mice that had received LTS samples experienced a significant decrease in tumor growth in contrast to mice with sample transplantation from STS or healthy control donors. On the other hand, the STS mice had larger tumors than mice with FMT from healthy control donor samples, suggesting that bacteria from LTS may have a protective effect against tumor growth and that PDAC-related bacteria might induce tumor development [44]. To characterize the microbial composition in PDAC, Zhou and colleagues tested 32 patients and compared their results to a healthy control group. They found a significant reduction in bacteria from the phylum *Firmicutes*, with butyrate-producing bacteria such as *Eubacterium rectale*, *Faecalibacterium prausnitzii* and *Roseburia intestinalis* being at the forefront, with a significant decrease in fecal butyrate, and a significant increase in phylum *Proteobacteria*, namely *Gammaproteobacteria*. In terms of species, Zhou et al. found 24 bacterial species that were enriched in PDAC samples with *Eubacterium rectale*, *Eubacterium ventrisum* and *Odoribacter splanchnicus* emerging as the most important biomarkers for differentiation between PDAC and healthy controls [169]. Currently, the M.D. Anderson Cancer Center is conducting early phase I trials to assess the safety, tolerability, and feasibility of FMT in patients with resectable PDAC. The expected completion date for these trials is by the end of 2023 (NCT04975217). Despite promising progress, several obstacles hinder the widespread utilization of FMT. Interindividual variations in physiology, immune system responses, dietary components, lifestyle, and genetics, in addition to donor-recipient relationship and microbiome complementarity contribute to variable outcomes following FMT [170, 171]. Consequently, further research is necessary to optimize clinical efficacy and improve the overall success rate of FMT procedures. By addressing these challenges, advancements can be made to enhance the clinical outcomes and ensure the safe and effective application of fecal microbial transplantation in medical practice.

### Challenges in microbiome-targeted therapies

The development of microbiota-based therapeutics is advancing due to synthetic biology and our improved understanding of host-associated microbial communities. However, numerous challenges must be addressed to effectively translate these advancements into clinical applications. Despite progress observed in animal models, the applicability of these findings to humans remains unexplored. The present knowledge gap in host interactions, immune responses, and interindividual variability in the composition and function of microbiome makes it difficult to develop standardized treatment strategies [99]. An understanding of the factors shaping host-associated microbial communities is vital for rational therapeutic design. In the context of microbiome-targeted therapies, the imperative is to advance biosensor and genetic circuit technologies.. Biosensors, designed to detect specific microbial biomarkers, and stable genetic circuits, intricately regulating gene expression, are pivotal for the success of autonomous cellular therapies. By improving these tools, we ensure treatments can sense and adapt to the microbiome’s changing conditions, making them more effective. Attention to regulatory, biocontainment, and safety is key for translating research to clinical applications. Overcoming challenges involves stable engraftment, suitable organisms, and efficient biosensors. Assessing circuit robustness and establishing safety frameworks ensure successful microbiome-targeted therapies [172]. Although current therapeutic approaches may appear rudimentary, ongoing research and engineering endeavors offer promising prospects for developing more effective and safer microbiota-based therapies, ultimately benefiting human health.

## Conclusions

The microbiome has received increasing attention as a potential biomarker for cancer diagnosis and assessing prognosis. The studies discussed have revealed significant associations between microbial composition and PC, with specific bacterial profiles identified in the GI tract, oral cavity, bile fluid, and tumor tissue of PC patients. These findings indicate the potential for non-invasive and easily accessible microbial markers that could broaden the screening criteria beyond high-risk individuals. The microbiome has also emerged as a promising potential therapeutic target in the prevention and treatment of PC. Traditional methods of PC treatment, such as surgery, chemotherapy, and radiation therapy, have limitations in terms of efficacy and may lead to significant side effects. Novel approaches, including personalized bacteriophage therapy, fecal microbial transplantation, prebiotics, and probiotics, have shown encouraging results in targeting the microbiome to improve PC outcomes. The modulation of the microbiome presents an exciting avenue for PC prevention and treatment. Targeting specific microbial populations and utilizing personalized therapeutic strategies may lead to improved outcomes and reduced side effects in PC patients. However, the results of these studies should be evaluated with caution due to several limitations and challenges.

Firstly, the microbiome is highly dynamic and influenced by several factors, including diet, lifestyle, and medication use. These factors can mask subtle differences in microbiome composition between healthy individuals and patients with PC, leading to false-positive or false-negative results [173]. Moreover, it is important to understand the role of the microbiome in the pathogenesis of PC, which needs further investigations. The studies conducted so far have only observed an association. However, the mechanistic link between the gut microbiome and PC needs to be further explored. The relatively small sample size and the heterogeneity of the patient populations studied previously have potentially led to the inconsistency of results [174]. Moreover, the control group in some of these studies has not been consistent, making it difficult to draw accurate conclusions [175]. Additionally, the lack of standardization in the techniques used to analyze microbiome composition has hampered the comparison of results across different studies, impeding the potential use of microbiome signatures in clinical practice [176].

Our understanding of the pathogenesis of PC and other GI tract organs is still growing as the link between gut microbiota and several neoplasms is being rigorously studied. Current studies have revealed the influence of oral, gastric, biliary, intrapancreatic, and intestinal microbial alterations on carcinogenesis through distinct pathways. Emerging evidence suggests the potential therapeutic effects of antibiotics, probiotics, bacteriophage therapy, and fecal microbial transplantation to regulate the microbial composition of the GI tract. Nonetheless, future studies should focus on larger patient populations and standardizing techniques to better understand the microbiome’s influence on cancer development and progression. More clinical studies are needed to further assess comorbidities, sampling methods, and variabilities among patients.

## Data Availability

Not applicable.

## Abbreviations

PC: Pancreatic cancer
PDAC: Pancreatic ductal adenocarcinoma
FPC: Familial pancreatic cancer
GI: Gastrointestinal
SCFA: Short chain fatty acid
STS: Short-term survival
LTS: Long-term survival
PCH: Pancreatic head cancer
PCB: Pancreatic body and tail cancer
NAT: Neoadjuvant therapy
TUBB: Tubulin, beta class I
FMT: Fecal material transplantation
